# The Cells and Extracellular Matrix of Human Amniotic Membrane Hinder the Growth and Invasive Potential of Bladder Urothelial Cancer Cells

**DOI:** 10.3389/fbioe.2020.554530

**Published:** 2020-11-09

**Authors:** Taja Železnik Ramuta, Urška Dragin Jerman, Larisa Tratnjek, Aleksandar Janev, Marta Magatti, Elsa Vertua, Patrizia Bonassi Signoroni, Antonietta Rosa Silini, Ornella Parolini, Mateja Erdani Kreft

**Affiliations:** ^1^Institute of Cell Biology, Faculty of Medicine, University of Ljubljana, Ljubljana, Slovenia; ^2^Centro di Ricerca E. Menni, Fondazione Poliambulanza Istituto Ospedaliero, Brescia, Italy; ^3^Department of Life Science and Public Health, Università Cattolica del Sacro Cuore, Rome, Italy; ^4^Fondazione Policlinico Universitario “Agostino Gemelli” IRCCS, Rome, Italy

**Keywords:** amniotic membrane, bladder cancer, tissue engineering, regenerative medicine, anticancer, amniotic epithelial cells, amniotic mesenchymal stromal cells, urothelial cancer cells

## Abstract

Bladder cancer is one of the most common cancers among men in industrialized countries and on the global level incidence and mortality rates are increasing. In spite of progress in surgical treatment and chemotherapy, the prognosis remains poor for patients with muscle-invasive bladder cancer. Therefore, there is a great need for the development of novel therapeutic approaches. The human amniotic membrane (hAM) is a multi-layered membrane that comprises the innermost part of the placenta. It has unique properties that make it suitable for clinical use, such as the ability to promote wound healing and decrease scarring, low immunogenicity, and immunomodulatory, antimicrobial and anticancer properties. This study aimed to investigate the effect of (i) hAM-derived cells and (ii) hAM scaffolds on the growth dynamics, proliferation rate, and invasive potential of muscle-invasive bladder cancer T24 cells. Our results show that 24 and 48 h of co-culturing T24 cells with hAM-derived cells (at 1:1 and 1:4 ratios) diminished the proliferation rate of T24 cells. Furthermore, when seeded on hAM scaffolds, namely (1) epithelium of hAM (e-hAM), (2) basal lamina of hAM (denuded; d-hAM), and (3) stroma of hAM (s-hAM), the growth dynamic of T24 cells was altered and proliferation was reduced, even more so by the e-hAM scaffolds. Importantly, despite their muscle-invasive potential, the T24 cells did not disrupt the basal lamina of hAM scaffolds. Furthermore, we observed a decrease in the expression of epithelial-mesenchymal transition (EMT) markers N-cadherin, Snail and Slug in T24 cells grown on hAM scaffolds and individual T24 cells even expressed epithelial markers E-cadherin and occludin. Our study brings new knowledge on basic mechanisms of hAM affecting bladder carcinogenesis and the results serve as a good foundation for further research into the potential of hAM-derived cells and the hAM extracellular matrix to serve as a novel bladder cancer treatment.

## Introduction

Approximately 549,400 people were diagnosed with bladder cancer worldwide in 2018 and as its incidence continues to increase, bladder cancer is classified among the five most common malignancies in industrialized countries (Zieger, 2008; Ferlay et al., 2015; Bray et al., 2018). Overall, men are more affected than women (3.2:0.9 ratio), and bladder cancer incidence and mortality are higher in high-income countries in comparison to low-income countries (Sanli et al., 2017; Cumberbatch et al., 2018).

There are two main clinical phenotypes of bladder cancer, namely non-muscle-invasive bladder cancer and muscle-invasive bladder cancer. At initial diagnosis, 75% of cases are non-muscle invasive bladder cancer and 25% are muscle-invasive bladder cancer. Moreover, 50–70% of patients with non-muscle-invasive bladder cancer have recurrences after surgical removal of the primary tumor, and 10–20% of those also progress to muscle-invasive bladder cancer (Soloway, 2013; Yun and Kim, 2013; Ye et al., 2014; Scarpato et al., 2015; Mari et al., 2017). The development of multifocal tumors dispersed in the urothelium is common. The multifocal tumors may be recognized already at first diagnosis of cancer, but more often arise after resection of the primary tumor (Habuchi, 2005; Kim et al., 2012). The development of muscle-invasive tumors (T2–T4 stage) presents a critical clinical step in carcinogenesis, which results in a significantly lower 5-year survival rate, and therefore demands more aggressive therapy (Mari et al., 2017; Sanli et al., 2017). Hence, it is imperative to develop new therapeutic approaches that will target bladder cancer cells with high proliferative and invasive potential, which will increase the success of muscle-invasive bladder cancer treatment.

The human amniotic membrane (hAM) is the innermost layer of the fetal membranes, which protects the embryo and later the fetus. hAM is composed of a monolayer of human amniotic epithelial cells (hAEC), the basal lamina and thick avascular stroma, which is further divided into the compact layer, a layer of human amniotic mesenchymal stromal cells (hAMSC) and a spongy layer (Rocha and Baptista, 2015). Moreover, the hAM and cells derived thereof have many properties which make it suitable for clinical use, such as low immunogenicity (Kubo et al., 2001; Szekeres-Bartho, 2002), immunomodulatory activity (Kronsteiner et al., 2011; Li et al., 2015; Magatti et al., 2015, 2017; Pianta et al., 2015) and antifibrotic activity (Tseng et al., 1999; Kim et al., 2000; Sant’anna et al., 2011), angiogenic and anti-angiogenic activity (Hao et al., 2000; Paeini-Vayghan et al., 2011; Niknejad et al., 2013a), promotion of epithelisation (Fukuda et al., 1999; Kim et al., 2000; Jin et al., 2015), antimicrobial activity (Talmi et al., 1991; Kjaergaard et al., 2001; Mencucci et al., 2006; Tehrani et al., 2013; Mao et al., 2017, 2018; Šket et al., 2019; Ramuta et al., 2020b) and also anticancer activity (Jiao et al., 2012; Magatti et al., 2012; Niknejad et al., 2013b, 2014, 2016; Mamede et al., 2014, 2015; Riedel et al., 2019).

The use of hAM in the clinic is increasing. While the hAM is already widely applied in ophthalmology, the potential of its use in various medical fields is demonstrated also by the increasing number of clinical trials reporting the utilization of hAM^1^. The use of hAM is relevant also in the field of urology, which has been demonstrated by several *in vitro* and *in vivo* studies. Previously we have shown that hAM as a scaffold enables the development of tissue-engineered urothelium, which is in molecular and ultrastructural properties comparable to native urothelium (Jerman et al., 2014). Other studies have already used the hAM for bladder (Shakeri et al., 2008; Adamowicz et al., 2016; Barski et al., 2017) and urethral reconstruction (Shakeri et al., 2009; Wang et al., 2014) in animal models. Moreover, hAM was also used for reconstructive surgery of the ureteral obstruction in patients with extensive ureteral strictures (Koziak et al., 2007) and reconstructive surgery of strictured urethra (Koziak et al., 2004).

The anticancer properties of hAM started gaining recognition in recent years. Magatti et al. (2012) and Bu et al. (2017) have demonstrated that hAMSC and hAEC induce a cell cycle arrest in the G0/G1 phase in several cancer cell lines. Moreover, several research groups have shown that hAM and its derivatives promote apoptosis in cancer cells and also reduce the viability and affect the metabolism of cancer cells (Jiao et al., 2012; Niknejad et al., 2013b, 2014; Mamede et al., 2014, 2015, 2016; Riedel et al., 2019). However, to the best of our knowledge, the effect of hAM on bladder cancer has not yet been extensively investigated. Therefore, the aim of our study was to investigate the effect of (i) hAM-derived cells and (ii) hAM scaffolds on the growth dynamics, proliferation, and invasive potential of T24 muscle-invasive bladder cancer cells.

## Materials and Methods

### Ethics Statement

The use of hAM was approved by the National Medical Ethics Committee of the Republic of Slovenia (decree numbers 43/12/09 and 0120-179/2018/5) and prepared according to the standard procedures (Mikek et al., 2004; Soncini et al., 2007; Jerman et al., 2014; Magatti et al., 2015; Cargnoni et al., 2018). Briefly, to prepare hAM scaffolds, 15 placentas were obtained with written informed consent at the time of elective cesarean sections from healthy volunteers, who were serologically negative for HIV, syphilis and hepatitis B and C. For hAM-derived cells (hAMSC and hAEC), human term placentas (*n* = 10) were collected from healthy women serologically negative for HIV, hepatitis B and C, after vaginal delivery or cesarean section at term after obtaining informed written consent according to the guidelines set by the Comitato Etico Provinciale of Brescia number NP 2243 (19/01/2016), Italy. For the preparation of primary urothelial cells, porcine urinary bladders were obtained from a local abattoir. The experiments were approved by the Veterinary Administration of the Slovenian Ministry of Agriculture and Forestry in compliance with the Animal Health Protection Act and the Instructions for Granting Permits for Animal Experimentation for Scientific Purposes (U34453-15/2013/2).

### Cell Cultures

In the experiments, the T24 cell line that originated from human invasive urothelial neoplasm (ATCC, United States) was used as a model of muscle-invasive bladder cancer cells (passages 5–30). The T24 cells were seeded at seeding density of 5 × 10^4^ cells/cm^2^ and cultured at 37°C and 5% CO_2_ in culture medium A-DMEM+F12 as described previously (Resnik et al., 2015, 2019; Višnjar et al., 2017). The culture medium consisted of 1:1 mixture of A-DMEM medium (Gibco, United States) and F12 (Sigma-Aldrich, United States), supplemented with 5% fetal bovine serum (FBS) (Invitrogen), 4 mM Glutamax (Gibco, United States), 100 μg/ml streptomycin, and 100 U/ml penicillin (Thermo Fisher Scientific, United States).

The CFPAC-1 cell line that originated from human pancreatic adenocarcinoma (ATCC, United States), Saos-2 cell line that originated from human bone osteosarcoma (kindly provided by the Centro Substrati Cellulari, Istituto Zooprofilattico of Brescia, Italy), and human fibroblasts isolated from a skin biopsy. Skin-derived fibroblasts were obtained after informed consent as described by Magatti et al. (2012), served as a control. CFPAC-1, Saos-2 and skin-derived human fibroblast cells were seeded at seeding density of 1 × 10^4^ cells/cm^2^ and cultured at 37°C and 5% CO_2_ in RPMI completed medium composed of RPMI 1640 medium (Sigma-Aldrich, United States) supplemented with 10% fetal bovine serum (FBS) (Sigma-Aldrich, United States), 2 mM L-glutamine (Sigma-Aldrich, United States), 100 U/mL penicillin (Sigma-Aldrich, United States), and 100 μg/mL streptomycin (Sigma-Aldrich, United States).

Primary normal porcine urothelial cell cultures (NPU cells) were established as described previously (Kreft et al., 2005; Višnjar et al., 2012) and cultured in culture medium consisting of equal parts of MCDB153 medium (Sigma-Aldrich, United States) and Advanced Dulbecco’s modified essential medium (Invitrogen, Life Technologies, United States), 0.1 mM phosphoethanolamine (Sigma-Aldrich), 15 μg/ml adenine (Sigma-Aldrich, United States), 0.5 μg/ml hydrocortisone (Sigma-Aldrich, United States), 5 μg/ml insulin (Sigma-Aldrich, United States), 4 mM glutamax (Gibco, United States), 100 μg/ml streptomycin and 100 U/ml penicillin (Thermo Fisher Scientific, United States). The NPU cells (passages 4–10) were plated on synthetic scaffolds (porous membrane, pore diameter 0.4 μm; BD Falcon, Corning, United States) at a seeding density of 1 × 10^5^ cells/cm^2^. When the NPU cells reached confluence, they were cultured in the culture medium without serum, but with a physiological calcium concentration of 2.7 mM. The NPU cells were maintained at 37°C and 5% CO_2_ for 3 weeks. The experiments were approved by the Veterinary Administration of the Slovenian Ministry of Agriculture and Forestry in compliance with the Animal Health Protection Act and the Instructions for Granting Permits for Animal Experimentation for Scientific Purposes.

### Isolation, Culture, Expansion and Characterization of hAEC and hAMSC

Isolation of hAEC and hAMSC from human term placentas was performed as described previously (Soncini et al., 2007; Magatti et al., 2015; Cargnoni et al., 2018). Briefly, the amniotic membrane was first manually separated from chorion and washed with 0.9% NaCl containing 100 U/ml penicillin and 100 μg/ml streptomycin (Sigma-Aldrich, United States) and 2.5 mg/ml amphotericin B (Sigma-Aldrich, United States). For hAMSC isolation, hAM was cut into pieces (3 cm × 3 cm), which were then sterilized by brief incubation in 0.9% NaCl and 2.5% Eso Jod (Esoform, Italy) and then in 500 U/ml penicillin, 500 μg/ml streptomycin, 12.5 μg/ml amphotericin B and 1.87 mg/ml Cefamezin (Teva Italia SrL, Italy). The pieces were then incubated for 9 min at 37°C in HBSS (Sigma-Aldrich, United States) containing 2.5 U/ml dispase (VWR International Srl, Italy), and digested in complete RPMI 1640 medium (Sigma-Aldrich, United States), supplemented with 0.94 mg/ml collagenase (Roche, Germany) and 10 μg/ml DNase (Roche, Germany) for 2.5–3.0 h at 37°C. The amniotic epithelium was then removed by centrifugation (3 min at 150 *g*) and hAMSC were passed through 100-μm and 70-μm cell strainers and then centrifuged. Then hAMSC (p0) were plated (seeding density 1 × 104 cells/cm^2^) and cultured at 37°C and 5% CO_2_ in DMEM complete medium supplemented with 10% heat-inactivated fetal bovine serum (FBS; Sigma-Aldrich, United States), 2 mM L-glutamine (Sigma-Aldrich, United States), 100 U/ml penicillin and 100 μg/ml streptomycin or cryopreserved in 10% DMSO (Sigma-Aldrich) supplemented with 90% FBS until use. When hAMSC reached confluence, they were subcultured at a seeding density 1 × 10^4^/cm^2^.

For hAEC isolation, hAM was cut into pieces (15 cm × 15 cm) which were incubated for 10 min at 37°C in PBS containing 0.5 mM EDTA and 100 U/ml penicillin and 100 μg/ml streptomycin, followed by a 5-min incubation in 1× trypsin/EDTA solution (Sigma-Aldrich, United States) at 37°C. The debris was discarded and the pieces were incubated for 10 min at 37°C in 1× trypsin-EDTA solution, followed by washing in PBS and they were digested a second time in 1× trypsin/EDTA for 10 min at 37°C. Cells from digestions were collected and centrifuged at 300 *g* for 10 min and the cell suspensions were filtered through a 70 μm cell strainer (BD Biosciences, United States), centrifuged again, plated (hAEC p0; seeding density 1.2 × 10^5^/cm^2^) and cultured at 37°C and 5% CO_2_ in DMEM/F12 complete medium. When hAEC reached confluence, they were subcultured at a seeding density 4 × 10^4^/cm^2^.

For phenotype evaluation, hAEC and hAMSC were trypsinized and then washed with FACS buffer (0.1% sodium azide (Sigma-Aldrich, United States) and 0.1% FBS (Sigma-Aldrich) in PBS), followed by a 20-min incubation at 4°C with anti-human fluorescein isothiocyanate- (FITC) or phyoerythrin- (PE) or allophycocyanin (APC)-conjugated antibodies or isotype controls (specified bellow) with 20 ml/ml polyglobin (Gammagard, United States) prepared in PBS with 1% BSA to block non-specific binding. After incubation, the cells were washed with FACS buffer and the dead cells were eliminated by the propidium iodide (PI) staining. The following clones of the monoclonal antibodies were used: CD73 (clone AD2), CD90 (5E10), CD45 (HI30), CD105 (266), CD13 (L138), CD66b (G10F5) (all from BD Biosciences, United States), and CD324 (67A4) (Miltenyi, Germany).

### Analysis of the Effect of hAMSC and hAEC on the Proliferation of T24 Cells

The effect of amniotic-derived cells on the proliferation of T24 cells was evaluated either in direct contact or with physical separation using transwell chambers, as described previously (Magatti et al., 2012). Briefly, for direct contact experiments, hAMSC and hAEC cells (p2) and control cells CFPAC-1, Saos-2, and skin-derived human fibroblast cells were plated in flat-bottom 96-well plates (Corning, United States) in RPMI complete medium at seeding density 1 × 10^4^ cells/well and 4 × 10^4^ cells/well. For non-contact experiments, hAMSC, hAEC, CFPAC-1, Saos-2, and skin-derived human fibroblast cells were plated in the upper compartment (0.4-μm pore, polycarbonate membranes, 96-well plates, Corning) of transwell chambers (1 × 10^4^ cells/transwell and 4 × 10^4^ cells/transwell of RPMI complete medium). hAMSC, hAEC, CFPAC-1, Saos-2, and fibroblast cells were gamma-irradiated to block their proliferation ensuring the maintenance of the correct ratio during co-culture (30 Gy for hAMSC, hAEC, and fibroblast dermal cells; 50 Gy for CFPAC-1, and Saos-2 cells). Therefore, any proliferation observed would be attributed only to the T24 cell line, which was added after 24 h to each well in direct contact with hAMSC, hAEC, CFPAC-1, Saos-2, and skin-derived human fibroblast cells or in the lower compartment of transwell chambers (seeding density 1 × 10^4^ cells/well in A-DMEM+F12 complete medium). After additional 24 and 48 h in culture, the proliferation of cancer cells was assessed by adding [^3^H]-thymidine (1 μCi/well; Perkin Elmer, Life Sciences, Belgium) for 16–18 h and then by harvesting the cells with a Filtermate Harvester (Perkin Elmer, Belgium). Thymidine incorporation was measured by using a microplate scintillation and luminescence counter (Top Count NXT; Perkin Elmer, Belgium).

### Preparation of hAM Scaffolds

Human amniotic membrane was manually separated from the chorion and washed with sterile phosphate-buffered saline (PBS), which contained antibiotics (50 μg/mL penicillin, 50 μg/mL streptomycin, 100 μg/mL neomycin) and an antimycotic (2,5 μg/mL amphotericin B). Then hAM was cut into pieces (4 cm × 4 cm) and cryopreserved at –80°C in MEM (Modified Eagle’s Medium; Gibco, United States) and glycerol (volume ratio 1:1) until use. After thawing at room temperature, hAMs were washed with sterile PBS (two times for 5 min) and A-DMEM+F12 culture medium (two times for 5 min). Next, the hAMs were fastened in membrane holders of 14 or 25 mm in diameter (CellCrown; Scaffdex, Finland) with the amniotic epithelium facing up (epithelial hAM: e-hAM; denuded hAM: d-hAM) or with the hAM stromal side facing up (stromal hAM: s-hAM). To obtain d-hAM, hAEC were removed by a 15 min incubation in the proteolytic enzyme thermolysin (1:50 in PBS; *Thermolysin from Bacillus thermoproteolyticus rokko*, Sigma-Aldrich, United States) at 37°C. After incubation, d-hAM scaffolds were rinsed with PBS and incubated for 15 min at room temperature on a shaker to remove cellular debris.

Before seeding, T24 cells were labeled with a fluorescent dye DiI (Invitrogen). Briefly, T24 cells were incubated in a mixture of the DiI dye (Thermo Fisher Scientific, United States) and A-DMEM+F12 culture medium (1:1000) and incubated at 37°C for 30 min. Next, T24 cells were rinsed with culture medium (three times for 5 min). Labeled T24 cells were seeded on hAM scaffolds and synthetic scaffolds (porous membrane, pore diameter 0.4 μm; BD Falcon, Corning, United States) at a seeding density of 5 × 10^4^ cells/cm^2^ and were then maintained at 37°C and 5% CO_2_ for 3 weeks. The culture medium was changed 3 times a week and each time before and after taking the images. After 3 weeks in culture, samples were further processed for light microscopy (paraffin sections and cryosections) and transmission and scanning electron microscopy.

### Analysis of Proliferation of T24 Cells Grown on hAM Scaffolds and Synthetic Scaffolds

To evaluate the growth of T24 cells on hAM scaffolds and synthetic scaffolds, T24 cells were observed with an inverted fluorescence microscope Eclipse TE300 (Nikon, Japan). Namely, on days 1, 2, 3, 7, 14, and 21 we took seven images of each scaffold at random positions. The area of the hAM scaffolds covered with T24 cells was evaluated with ImageJ software (Schneider et al., 2012), measuring this area as a percentage of total field view area.

The proliferation rate of T24 cells grown on hAM and synthetic scaffolds was analyzed by labeling the proliferating cells with the Click-it Plus EdU Alexa Fluor 488 Imaging Kit (Thermo Fisher Scientific, United States) and subsequent analysis with ImageJ software. Briefly, 24 h before the analysis the T24 cells grown on hAM and synthetic scaffolds were incubated in the culture medium A-DMEM+F12 supplemented with 10 μM 5-ethynyl-2 ′-deoxyuridine (EdU). To evaluate the proliferation of hAM-derived cells, the e-hAM scaffolds alone were incubated in the culture medium A-DMEMCF12 supplemented with 10 μM EdU. After 24 h, hAM scaffolds and synthetic scaffolds were fixed for 15 min with 4% formaldehyde at room temperature, washed three times with 3% BSA/PBS, permeabilized with 0.5% Triton-X-100 in PBS and incubated at room temperature for 20 min. After permeabilization, samples were washed three times with 3% BSA/PBS. To stain the proliferating T24 cells, Click-it Plus reaction buffer containing AlexaFluor picolyl azide was prepared according to the manufacturer’s instructions and samples were incubated with the reaction buffer at room temperature for 30 min. Afterward, samples were washed three times with 3% BSA/PBS, nuclei were stained with DAPI and samples were examined with a fluorescence microscope AxioImager.Z1 equipped with ApoTome (Zeiss, Germany). Furthermore, 10 images for each sample were taken and the ratio of proliferating cells was analyzed with the ImageJ software.

### Light Microscopy and Immunofluorescence Labeling

To prepare paraffin sections, hAM scaffolds with T24 cells were fixed with 4% formaldehyde (w/v) overnight and then paraffin sections were prepared as previously described (Jerman et al., 2014). Briefly, samples were dehydrated through a graded series of ethanol into xylene, followed by embedment in paraffin wax and preparation of paraffin sections. Then these were stained with Periodic Acid Schiff stain (PAS) for neutral proteoglycans.

For immunofluorescence, hAM scaffolds and synthetic scaffolds with T24 cells were fixed in (a) 4% formaldehyde (w/v) for 15 min, washed in PBS for 30 min, embedded in OCT and then cryosections were prepared, and (b) in ice-cold absolute ethanol for 20 min at room temperature. After blocking in 3% BSA/PBS for 45 min, samples were incubated in polyclonal anti-collagen IV antibody (dilution 1:200; ab6586, Abcam, United Kingdom), rabbit polyclonal anti-N-cadherin antibody (dilution 1:100; ab18203; Abcam, United Kingdom), rabbit polyclonal anti-occludin antibody (dilution 1:30; 71–1500, Invitrogen, Thermo Fisher Scientific, United States), mouse polyclonal anti-E-cadherin antibody (dilution 1:20; 610182 BD-Pharmingem, United States) and rabbit polyclonal anti-Snail + Slug antibody (dilution 1:1000; ab180714, Abcam, United Kingdom) overnight at 4°C. As a positive control for occludin and E-cadherin stainings, the NPU cells were analyzed. The next day samples were washed in PBS and then incubated in goat anti-rabbit and goat anti-mouse IgG conjugated with Alexa Fluor 488 or Alexa Fluor 555 for 2 h at room temperature (dilution 1:400; Molecular Probes, Invitrogen, Thermo Fisher Scientific, United States). Afterward, DAPI was applied to stain nuclei. Samples were examined with the inverted fluorescence microscope Eclipse TE300 (Nikon, Japan) and a fluorescence microscope AxioImager.Z1 equipped with ApoTome (Zeiss, Germany).

### Western Blot Analysis

For Western blot analysis, T24 cells grown on hAM and synthetic scaffolds were lysed in ice-cold extraction buffer, comprised of a deionized H_2_0, 0.5 M TRIS HCl, 10% Sodium Dodecyl Sulfate (SDS) supplemented with a cocktail of protease and phosphatase inhibitors (Thermo Fisher Scientific, United States). Total protein quantity was determined using the Pierce BCA Protein Assay Kit (Thermo Fisher Scientific, United States). Protein extracts (50 μg) were separated using 4–20% Novex WedgeWell Tris-Glycine Gels (Invitrogen, Carlsbad, CA, United States) and then transferred onto the nitrocellulose membranes (Sigma-Adrich, St. Louis, MO, United States). The membranes were blocked with 5% skim milk in 0.1% Tris-buffered saline/Tween 20 (TBS-T) for 1 h at room temperature. Next, the membranes were incubated overnight with primary antibodies: anti-N-cadherin (dilution 1:1000; ab18203, Abcam, United Kingdom), anti-Snail + Slug (dilution 1:1000; ab180714, Abcam, United Kingdom), anti-actin (dilution 1:2000; A2066, Sigma-Adrich, United States), anti-collagen IV (dilution 1:1000; ab6586, Abcam, United Kingdom) at 4°C with gentle shaking. Next day, the membranes were washed with TBS-T and incubated with HRP-conjugated goat anti-rabbit IgG antibody (dilution 1:1000; A6154, Sigma-Adrich, United States) for 1 h at room temperature with gentle shaking. Finally, SuperSignal West Pico Chemiluminescent Substrate (Thermo Scientific, Waltham, MA, United States) was used to visualize the bands. Densitometric analysis was performed using the Image J software. Actin served as a loading control.

### Transmission and Scanning Electron Microscopy

Human amniotic membrane scaffolds and synthetic scaffolds with T24 cells were prepared for transmission and scanning electron microscopy as described previously (Višnjar et al., 2012; Jerman et al., 2014). Briefly, samples for scanning electron microscopy were fixed with 2% formaldehyde and 2% glutaraldehyde in 0.2 M cacodylate buffer, pH 7.4 for 3 h at 4°C. Fixation was followed by overnight rinsing in a 0.2 M cacodylate buffer and then by postfixation in 1% (w/v) osmium tetroxide in the same buffer for 2 h at room temperature. Samples were then dehydrated in a graded series of alcohol, followed by acetone and immersed in hexamethyldisilazane and dried at room temperature. Afterward, the samples were sputtered with gold and examined by a scanning electron microscope (Tescan Vega3). Samples for transmission electron microscopy (TEM) were fixed with 3% formaldehyde and 3% glutaraldehyde in 0.1 M cacodylate buffer for 3 h at 4°C. Fixation was followed by overnight rinsing 0.1 M cacodylate buffer and then by postfixation with 2% (w/v) osmium tetroxide for 1 h at room temperature. Samples were then dehydrated in a graded series of ethanol and embedded in Epon (Serva Electrophoresis). Ultrathin sections were contrasted with uranyl acetate and lead citrate and examined by a transmission electron microscope (Philips CM100).

### Statistical Analyses

All data were calculated from 3 to 5 biological samples of hAM and at least 3–10 technical repeats for each biological sample. Data are presented as mean ± SE. Statistical analysis was performed using a two-tailed Student’s *t*-test or two-way analysis of variance (ANOVA) when appropriate, adjusted by Tukey’s or Sidak’s method. Statistical analysis was performed using Prism 8 (GraphPad Software, La Jolla, CA, United States). *p*-values of <0.05 were considered statistically significant.

### Data Availability

The authors declare that all data supporting the findings of this study are available within the manuscript. All information about the materials and methods used are available also in the Protocols.io database (Ramuta et al., 2020a).

## Results

### Effect of hAM-Derived Cells on the Proliferation of Muscle-Invasive Bladder Cancer Cells

**FIGURE 1:**
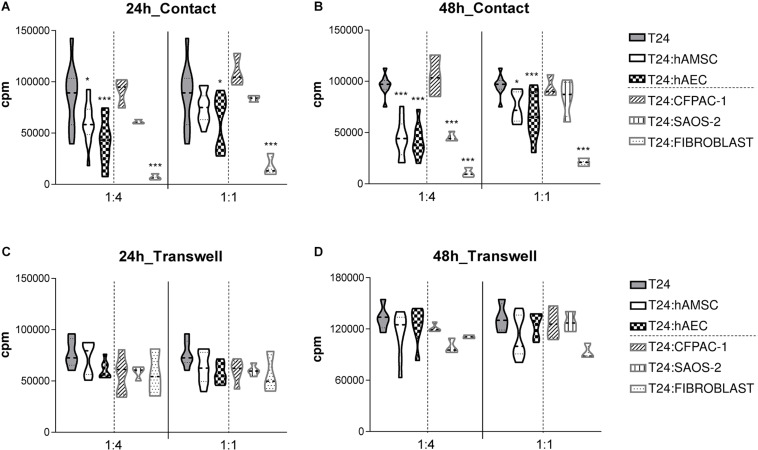
hAMSC and hAEC decrease the proliferation of T24 cells when grown in direct co-culture for 24 and 48 h. T24 cells were cultured alone or in the presence of different gamma-irradiated cell types (hAMSC or hAEC, CFPAC-1, Saos-2, dermal fibroblast cells). Cultures were performed in both contact **(A,B)** and transwell settings **(C,D)** at T24:gamma-irradiated cell ratios of 1:1 and 1:4. After 24 h **(A,C)** or 48 h **(B,D)** T24 cell proliferation was assessed by [3H]-thymidine incorporation. Data are expressed as violin plot of more than four independent experiments for each condition, five to nine biological samples of hAEC and hAMSC were used. *p < 0.05, **p < 0.01; ***p < 0.001.

To test the effect of hAM-derived cells on the proliferation of T24 cells, we grew them in direct co-culture with hAEC and hAMSC cells, namely in 1:4 and 1:1 T24:hAM cell ratios. Then we analyzed the proliferation of T24 cells 24 h (Figure 1A) and 48 h (Figure 1B) after co-culture. Our results show that hAEC and hAMSC decrease T24 cell proliferation when cultured in direct contact with T24 cells and stronger effects were observed after 48 h of culture and at a T24:hAMSC/hAEC ratio of 1:4. Indeed, after 24 h in co-culture, hAMSC decreased the proliferation of T24 cells on average by 31% (1:4 ratio; *p* < 0.05) and 11% (ratio 1:1), respectively. Moreover, when grown in co-culture with hAEC, the percentage of proliferating T24 cells significantly decreased by approximately 52% (1:4 ratio; *p* < 0.001) and 30% (1:1 ratio; *p* < 0.05), respectively (Figure 1A). After 48 h in co-culture, hAMSC inhibited the proliferation of T24 cancer cells by 53% (1:4 ratio; *p* < 0.001) and 21% (1:1 ratio; *p* < 0.05), while hAEC inhibited T24 cell proliferation by 57% (1:4 ratio; *p* < 0.001) and 31% (1:1 ratio; *p* < 0.001) (Figure 1B).

To investigate if any anti-proliferative effects were observed also in absence of cell-contact, co-cultures were physically separated by a transwell system. In this non-contact setting, we did observe a slight decrease of T24 proliferation after both 24 h (Figure 1C) and 48 h (Figure 1D) of culture, suggesting the need of cell-contact in the hAM-derived cell reduction of T24 cell proliferation.

In addition, to test whether these results are specific to human hAM-derived cells, and whether the co-culture condition alone or rather the decreased surface area available for cell growth, is responsible for the decrease in proliferation of T24 cells, we cultured T24 with the cystic fibrosis pancreatic adenocarcinoma (CFPAC-1) and Saos-2 cells which have an epithelial-like morphology similar to hAEC, or with skin-derived fibroblasts which are stromal cells similar to hAMSC. Co-culture with CFPAC-1 cells for 24 h (Figure 1A) and 48 h (Figure 1B), at both T24:CFPAC-1 of 1:4 and 1:1, had no statistically significant effect on T24 cell proliferation Similarly, at both ratios tested, Soas cells had no statistically significant effect on T24 cell proliferation when cultured in contact with T24 cells for 24 h (Figure 1A). After 48 h of co-culture, we observed a reduction of T24 cancer cell proliferation only when co-culture with Saos-2 cells was performed at 1:4 ratio (inhibition of 53%; *p* < 0.001), but not when cultured at 1:1 ratio (reduction of 11%; not significant) (Figure 1B). Instead, the stromal fibroblast cells were able to inhibit the proliferation of T24 cells after 24 h (Figure 1A) and 48 h (Figure 1B), at both ratios tested.

When cultured in the transwell system, CFPAC-1, Saos-2, or skin-derived human fibroblast cells were unable to decrease T24 cell proliferation at both ratios tested and both after 24 h (Figure 1C) and 48 h (Figure 1D).

### Effect of the Cells and Extracellular Matrix in hAM Scaffolds on the Growth Dynamics and Proliferation of Muscle-Invasive Bladder Cancer Cells

**FIGURE 2:**
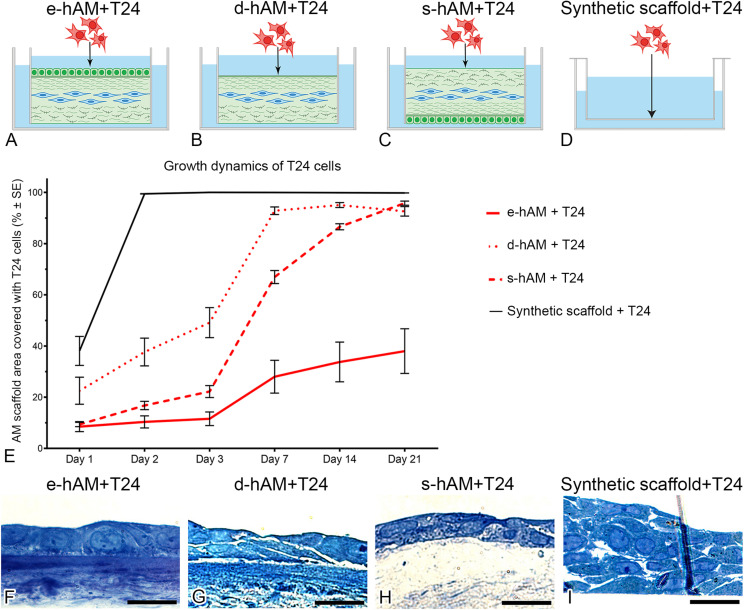
hAM scaffolds change the growth dynamic of T24 cells. T24 cells were seeded on **(A)** the hAM epithelium (e-hAM), **(B)** denuded hAM (d-hAM) without hAM epithelium but preserved basal lamina, **(C)** hAM stroma (s-hAM) of inverted hAM, and **(D)** on the synthetic scaffold. When grown on the hAM scaffolds, T24 cells growth was restricted. The growth restriction effect was pronounced especially by the e-hAM scaffold, followed by s-hAM and d-hAM scaffolds, especially in the first 3 days of culture **(E)** (*p* < 0.001). After 3 weeks in culture, T24 cells grown on the e-hAM **(F)** and s-hAM **(H)** scaffolds formed a 1–2 layered epithelium and on the d-hAM scaffolds they formed a 1–3 layered epithelium **(G)**. On the synthetic scaffolds T24 cells grew in 2–6 layers **(I)**. Scale bars: 50 μm.

T24 cells were seeded on the hAM and synthetic scaffolds (Figures 2A–D), and cultured 3 weeks. First, we monitored the growth of T24 cells by measuring the area of the scaffold covered by T24 cells. Our results show that the growth of T24 cells was diminished by all hAM scaffolds. The strongest growth inhibition was observed by the e-hAM scaffold, followed by the s-hAM scaffold. The first 3 days of culture were the most significant in terms of hAM scaffold potential to inhibit the growth of T24 cells. Namely, after 1 day in culture, T24 cells covered 38.09 ± 5.7% of the synthetic scaffolds, but only 8.49 ± 2.0% of the e-hAM scaffolds, 22.51 ± 5.3% of the d-hAM scaffolds and 9.33 ± 0.8% of the s-hAM scaffolds. After 3 days in culture, T24 cells overgrew (100%) the synthetic scaffolds, but they covered only 11.57 ± 2.7% of the e-hAM scaffolds, 47.13 ± 5.9% of the d-hAM scaffolds and 22.20 ± 2.3% of the s-hAM scaffolds. Even after 3 weeks in culture the T24 cells maintained confluent growth (100%) on the synthetic scaffolds but reached only 38.01 ± 8.7% coverage of the e-hAM scaffolds, 92.61 ± 1.85% of the d-hAM scaffolds and 95.81 ± 0.82 of the s-hAM scaffolds (Figure 2E). The dynamic of growth of T24 cells on the synthetic scaffolds was significantly different when compared to the hAM scaffolds (*p* < 0.001). Moreover, on the e-hAM and s-hAM scaffolds the T24 cells grew in a monolayer or, in rare exceptions, in two layers (Figures 2F,H). On the d-hAM scaffolds the T24 cells grew in 1–3 layers (Figure 2G), and when cultured on the synthetic scaffolds they grew in 2–6 layers (Figure 2I).

**FIGURE 3:**
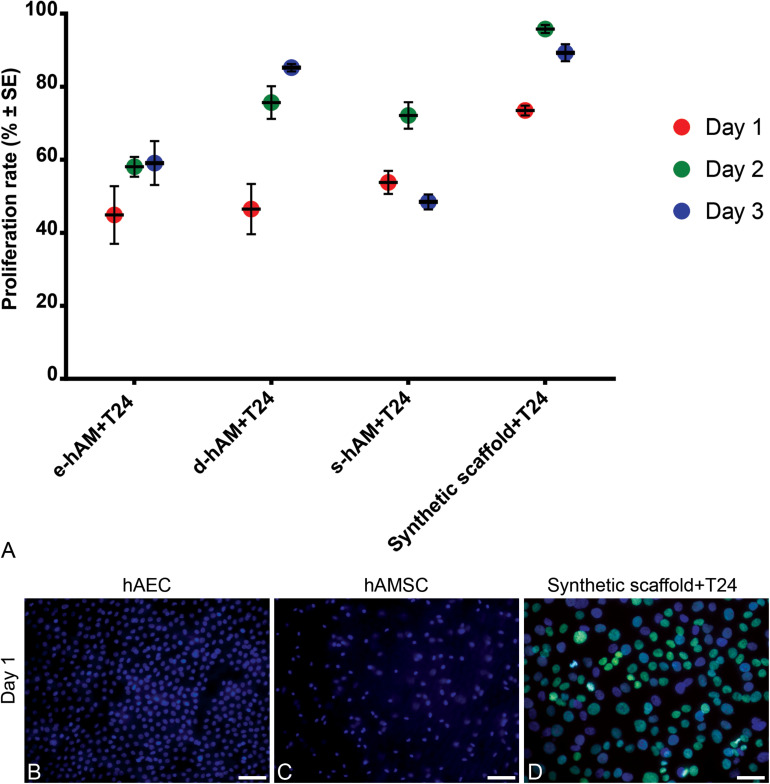
hAM scaffolds diminish the proliferation rate of T24 cells. **(A)** When grown on hAM scaffolds, the proliferation rate of T24 cells grown on the hAM scaffolds was diminished in comparison to the proliferation rate of T24 cells grown on the synthetic scaffolds. The proliferation rate was most diminished on day 1 in T24 cells grown on e-hAM and d-hAM scaffolds, and on day 3 in T24 cells grown on the s-hAM scaffolds. Data were obtained from at least 3 biological repeats and 20–30 technical repeats. **(B,C)** hAEC and hAMSC in e-hAM scaffolds did not proliferate, therefore, the rate proliferation of T24 cells grown on hAM scaffolds can be attributed to the T24 cells alone. **(D)** The proliferating T24 cells grown on the synthetic scaffold are labeled green. Green – EdU, incorporated in the DNA of T24 cells; blue – DAPI. Scale bars: 50 μm.

Furthermore, we analyzed the proliferation rate of T24 cells grown on hAM scaffolds and the synthetic scaffolds. All hAM scaffolds significantly changed proliferation rates of T24 cells on days 1 and 2, while on day 3 only e-hAM and s-hAM scaffolds significantly changed proliferation rates (*p* < 0.05; Table 1 and Figure 3A). In comparison to the proliferation rate on the synthetic scaffolds, the proliferation rate of T24 cells grown on e-hAM scaffolds was diminished for 29% on day 1, 38% on day 2, and 30% on day 3. When grown on the d-hAM scaffolds, the proliferation rate was diminished for 27% on day 1, for 20% on day 2, and 4% on day 3. Similarly, when grown on the s-hAM scaffolds the proliferation rate decreased for 18% on day 1, for 24% on day 2, and 41% on day 3 (Table 1 and Figure 3). Furthermore, analysis of e-hAM scaffolds without T24 cells showed that hAEC and hAMSC did not proliferate (Figures 3B,C), which confirmed that using this assay we quantified the proliferation of only T24 cells.

**TABLE 1:** hAM scaffolds diminish the proliferation rate of T24 cells.

hAM scaffolds with T24 cells	The percentage of T24 cell proliferation on different hAM scaffolds ± SE		
	Day 1	Day 2	Day 3
e-hAM+T24	44.9 ± 7.9	58.1 ± 2.7	59.1 ± 6.0
d-hAM+T24	46.5 ± 6.9	75.7 ± 4.5	85.2 ± 1.0
s-hAM+T24	53.8 ± 3.1	72.1 ± 3.6	48.5 ± 2.1
Synthetic scaffold+T24	73.5 ± 1.4	95.8 ± 1.1	89.3 ± 2.3

*Mean values (%) ± SE are shown. The percentage of proliferation shows the ratio of the population of T24 cells grown on hAM scaffolds or on the synthetic scaffolds that proliferated on days 1, 2, or 3.*

### Effect of hAM Scaffolds on the Invasive Potential of Bladder Cancer Cells and Their Ultrastructure

**FIGURE 4:**
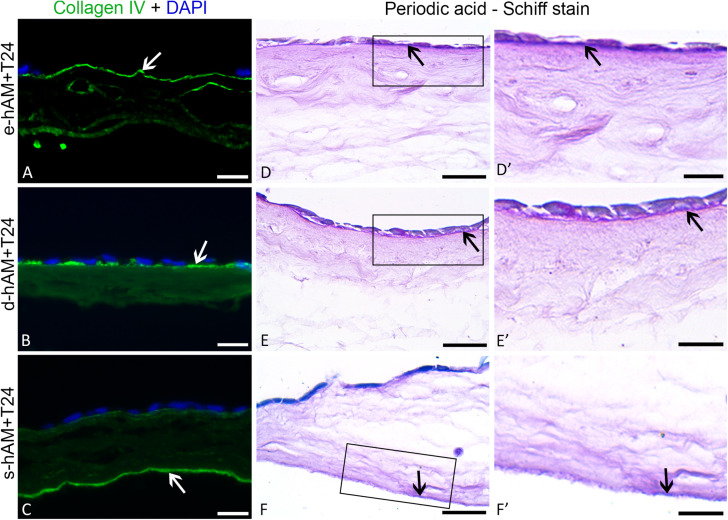
Muscle-invasive T24 cells do not disrupt the basal lamina ofhAM. T24 cells failed to disrupt the basal lamina (white and black arrows) of the hAM scaffolds and to invade into the hAM extracellular matrix **(A–F’)**. Images show T24 cells on hAM scaffolds after 3 weeks in culture. Frames mark enlarged areas shown in **(D’–F’)**. Green – collagen IV, DAPI – nuclei; arrows – basal lamina. Scale bars: **(A–C)** 50 μm, **(D–F)** 25 μm, and **(D’–F’)** 10 μm.

Since T24 cells originate from the muscle-invasive bladder cancer, our next goal was to evaluate whether T24 cells can have any effect on hAM basal lamina. Therefore, we immunolabelled collagen IV and performed a Periodic Acid-Schiff reaction (PAS). Our results show that hAM basal lamina remained intact even after 3 weeks in culture with T24 cells in all hAM scaffolds. The T24 cells did not invade the extracellular matrix of any of the hAM scaffolds (Figures 4A–F’).

**FIGURE 5:**
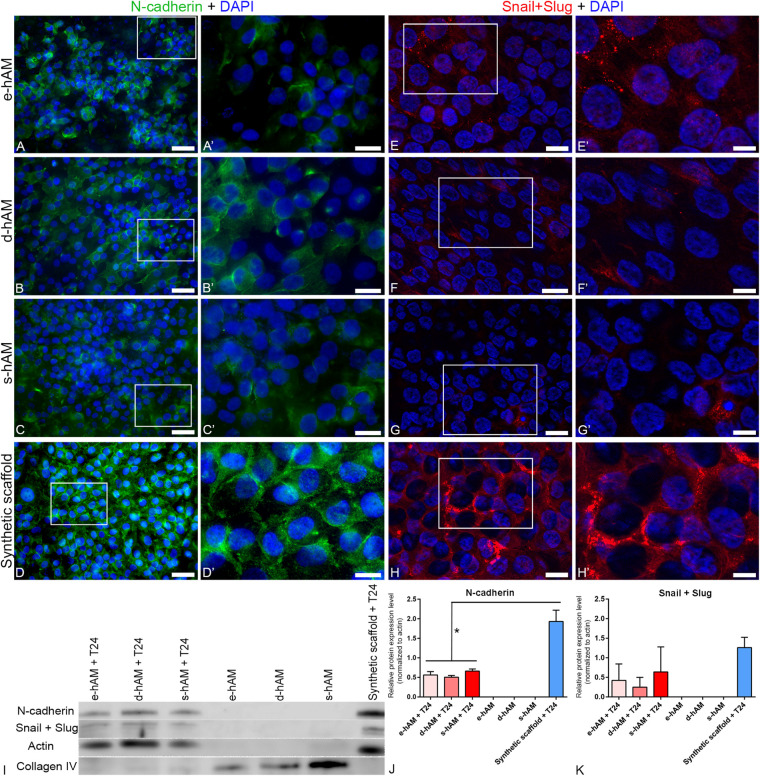
hAM scaffolds affect the expression of N-cadherin and Snail+Slug in T24 cells. **(A–C,A’–C’)** Individual T24 cells grown on hAM scaffolds expressed N-cadherin in the lateral plasma membrane of adjacent cells, however, in most cells N-cadherin was dispersed in the cytoplasm. **(D,D’)** N-cadherin was in T24 cells grown on synthetic scaffolds mainly distributed in a continuous line at the cell periphery. Images show T24 cells on hAM scaffolds and synthetic scaffolds after 3 weeks in culture. **(E–G’)** Diminished expression of Snail+Slug in T24 cells grown on hAM scaffolds. Namely, Snail+Slug were expressed mostly in the cytoplasm. **(H,H’)** In T24 cells grown on synthetic scaffolds Snail+Slug were mostly expressed in the perinuclear are and also in the cytoplasm and in the nucleus. Shown is the lowest expression of N-cadherin **(A–C’)** observed in T24 cells on hAM-scaffolds and the representative expression of N-cadherin in T24 cells grown on synthetic scaffolds **(D,D’)**. Shown is the representative expression of Snail+Slug in T24 cells grown on hAM and synthetic scaffolds **(E–H’)**. **(I–K)** Western blot analysis of N-cadherin, Snail+Slug, actin and collagen IV expression in the T24 cells grown on hAM scaffolds and synthetic scaffolds and in the hAM scaffolds alone. Frames mark enlarged areas shown in **(A’–H’)**. Green – N-cadherin, red – Snail+Slug, DAPI – nuclei. Scale bars: **(A–D)** 50 μm, **(E–H)** 20 μm, and **(A’–H’)** 10 μm. *p < 0.05.

To further evaluate the invasive potential of T24 cells grown on hAM scaffolds, we examined the expression of N-cadherin, a hallmark of epithelial-to-mesenchymal transition (EMT), which results in the acquisition of an aggressive tumor phenotype with enhanced migratory and invasive capacity (Mrozik et al., 2018). We have demonstrated overall diminished expression of N-cadherin in T24 cells grown on hAM scaffolds. Namely, while on synthetic scaffold all T24 cells expressed N-cadherin, on hAM scaffold only a part of T24 cells expressed N-cadherin (Figures 5A–D’). Furthermore, the expression of N-cadherin varied between different hAM scaffolds. When T24 cells were grown on e-hAM scaffolds, N-cadherin expression was dispersed in the cytoplasm (Figures 5A,A’), rarely N-cadherin was expressed in the lateral plasma membrane on borders between the adjoining cells (Figures 5A,A’). A similar expression of N-cadherin was observed in T24 cells on d-hAM scaffolds (Figures 5B,B’). In T24 cells grown on the s-hAM scaffolds expression of N-cadherin was strongly diminished and only a few cells expressed N-cadherin in the lateral plasma membrane or/and in the cytoplasm (Figures 5C,C’). On the contrary, the expression of N-cadherin in T24 cells grown on the synthetic scaffolds was strong and localized predominantly in the lateral plasma membrane of adjacent cells (Figures 5D,D’). Furthermore, we also performed a western blot analysis, which showed a significant decrease in expression levels of N-cadherin in T24 cells grown on the hAM scaffolds in comparison to the T24 cells grown on synthetic scaffolds (Figures 5I,J).

Next, we examined the expression of transcription factors Snail and Slug, which are markers of EMT (Wu et al., 2013) and are also connected with muscle invasion and metastasis in bladder cancer. Our results show overall diminished expression of Snail and Slug in T24 cells grown on the hAM scaffolds (Figures 5E–G’) in comparison to the T24 cells grown on synthetic scaffolds (Figures 5H,H’). Namely, Snail and Slug were in T24 cells grown on hAM scaffolds detected mostly in the cytoplasm, while they were in T24 cells grown on synthetic scaffolds detected in mostly in the perinuclear area and also in the cytoplasm and nuclei. Furthermore, we also performed a western blot analysis, which confirmed the trend of diminished expression of Snail in Slug in T24 cells grown of hAM scaffolds observed by the immunofluorescence staining (Figures 5I,K). With the aim of selecting appropriate loading control for data normalization, we investigated the expression of actin in all of our samples. Western blot analysis showed no signal for actin in the e-hAM, d-hAM and s-hAM scaffolds without the T24 cells. This result suggests that hAEC and/or hAMSC in the cryopreserved hAM, which was used to prepare hAM scaffolds, are damaged and characterized with low viability after 3 week period of cultivation. To confirm the presence of proteins in hAM scaffolds we additionally performed a western blot analysis of collagen IV, which showed that most of the proteins in the hAM scaffolds alone without the T24 cells are attributed to the extracellular matrix compared to the hAM scaffolds with T24 cells, in which most of the proteins are attributed to the cancer cells.

**FIGURE 6:**
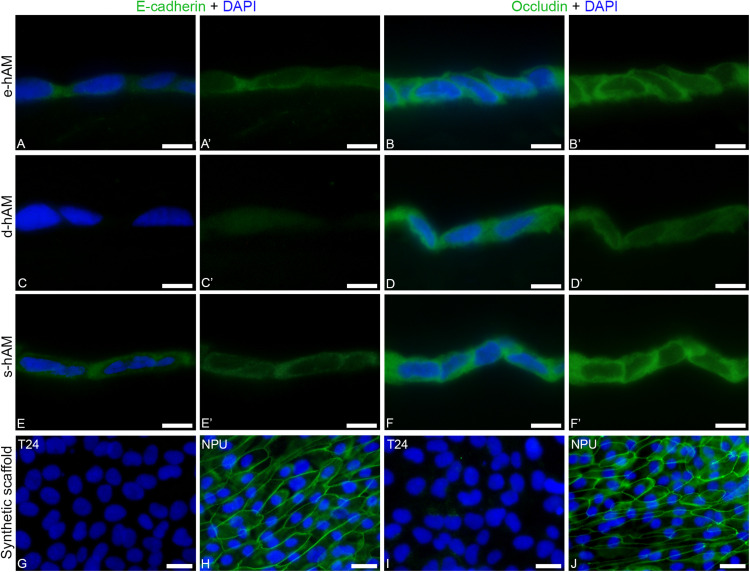
Individual T24 cells grown on hAM scaffolds expressed E-cadherin and occludin. **(A,A’,B,B’)** Individual T24 cells grown on e-hAM scaffolds expressed E-cadherin and occludin mainly in the cytoplasm. **(C,C’,D,D’)** The T24 cells grown on d-AM scaffolds did not express E-cadherin, while individual T24 cells grown on the d-AM scaffolds did express occludin. **(E,E’,F,F’)** The expression of E-cadherin and occludin was most prominent in the individual T24 cells grown on s-hAM scaffolds, in which both proteins were mainly dispersed in the cytoplasm and in some cases occludin was expressed on borders between adjoining cells. **(G,I)** T24 cells grown on synthetic scaffolds do not express E-cadherin nor occludin. **(H,J)** The strong expression of E-cadherin and occludin in normal porcine urothelial cells. Images show T24 cells on hAM scaffolds and synthetic scaffolds after 3 weeks in culture and normal porcine urothelial (NPU) cells on synthetic scaffolds after 4 weeks in culture. Green – Occludin, E-cadherin; DAPI – nuclei. Scale bars: 20 μm.

Next, we examined the expression of epithelial markers, such as E-cadherin and occludin and performed ultrastructural analysis of T24 cells. We found that individual T24 cells expressed E-cadherin when grown on e-hAM and s-hAM scaffolds (Figures 6A,A’,E,E’). E-cadherin was mainly dispersed in cytoplasm, in few cases T24 cells grown on s-hAM scaffolds have localized E-cadherin on the cell periphery (Figures 6A,A’,E,E’). Contrary, none of the T24 cells grown on the synthetic scaffold expressed E-cadherin (Figures 6C,C’G). Moreover, in normal porcine urothelial cells (Figure 6H) E-cadherin was expressed on borders between adjoining cells.

Similarly, individual T24 cells grown on hAM scaffolds also expressed occludin (Figures 6B,B’,D,D’,F,F’). On the hAM scaffolds the occludin was expressed mainly in the cytoplasm of individual T24 cells, and this expression was higher in T24 cells grown on the e-hAM and s-hAM scaffolds (Figures 6B,B’,F,F’) than in the T24 cells grown on the d-hAM scaffolds (Figures 6D,D’). Furthermore, the individual T24 cells grown on the e-hAM and s-hAM scaffolds expressed occludin on borders between adjoining cells (Figures 6B,B’,F,F’). None of the T24 cells grown on the synthetic scaffold (Figure 6I) expressed occludin. Importantly, such localization of occludin shows some similarities with the expression of occludin in normal porcine urothelial cells (Figure 6J).

**FIGURE 7:**
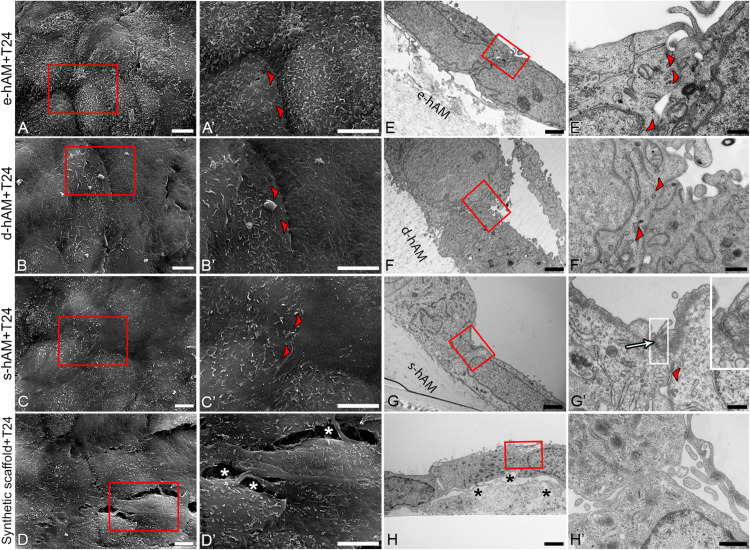
Ultrastructure of T24 cells on hAM and synthetic scaffolds. Scanning **(A–D’)** and transmission **(E–H’)** electron microscopy images are represented. T24 cells are tightly attached (arrowheads) to each other on hAM scaffolds in contrast to synthetic scaffolds where large intercellular spaces were observed (white and black asterisks, **D’,H**). Tight junction is formed between T24 cells on s-hAM (arrow, **G’**). Images show T24 cells on hAM scaffolds and synthetic scaffolds after 3 weeks in culture. Large inset framed with white lines show 200% enlarged image of corresponding small white framed inset **(G’)**. Red frames mark enlarged areas shown in **(A’–D’)** and **(E’–H’)**, respectively. Scale bars: **(A–D,A’–D’)** 5 μm; **(E–H)** 2 μm; **(F’,H’)** 600 nm, **(E’)** 400 nm, and **(G’)** 100 nm.

The ultrastructural analysis of T24 cells on hAM and synthetic scaffolds showed that in general, T24 cells were more tightly attached to each other when grown on the hAM scaffolds compared to synthetic scaffolds, where large intercellular spaces were observed (Figure 7). Moreover, T24 cells occasionally formed tight junctions when grown on s-hAM (Figures 7G,G’), thus corroborating occludin expression on borders between adjoining T24 cells demonstrated by immunolabeling (Figures 6E,E’). On all scaffolds, the T24 had large nuclei with a relatively small amount of cytoplasm. In few T24 cells on synthetic scaffolds, the amount of granular endoplasmic reticulum (GER) was prominent, however, altogether the significant ultrastructural differences in GER, Golgi apparatus, and mitochondria between T24 cells on different scaffolds were not noticed. The surface of T24 cells grown on s-hAM scaffolds exhibit relatively smooth surface, while on the synthetic scaffold the surface of T24 cells was shaped in irregular protrusions and microvilli (Figures 7E–H’).

## Discussion

Despite high initial response rates to multiagent chemotherapy, the median survival for patients with muscle-invasive bladder cancer is 15 months and the 5-year survival rate is only 15% (Von Der Maase et al., 2000, 2005), because many patients experience disease relapse despite effective therapy. Since a poor prognosis has often been attributed to resistance to chemotherapy and radiation therapy (Alfred Witjes et al., 2017; Mari et al., 2017), there is a great need for the development of new therapeutic approaches for the treatment of bladder cancer.

### hAM-Derived Cells Reduce the Proliferation of Muscle-Invasive Bladder Cancer Cells

Herein, we show that both hAMSC and hAEC reduce the proliferation of T24 cells, when cultured in direct contact with T24 cells and especially when cultured at a 1:4 T24:hAM cell ratio.

Our results show some heterogeneity between different biological samples, which can be attributed to placenta donor heterogeneity and also to heterogeneity among cells derived from different parts of the hAM. As Centurione et al. (2018) have already shown, hAEC isolated from different areas of hAM are a heterogeneous cell population and since hAEC and hAMSC used in our experiments were isolated from the whole hAM, some heterogeneity between different cell populations can be attributed to this as well (Centurione et al., 2018). In addition, different regions of the placenta have been shown to possess different metabolic activities, which could in turn also contribute to the heterogeneity observed (Banerjee et al., 2015, 2018; Centurione et al., 2018).

In addition to hAM-derived cells, T24 cells were co-cultured also with the CFPAC-1, Saos-2 and skin-derived human fibroblast cells. CFPAC-1 cells did not decrease the rate of proliferation of T24 cells in all the co-culture conditions tested, thus suggesting that the decrease in T24 cell proliferation observed with hAM-derived cells could be attributed to an active mechanism and not to medium exhaustion of nutrients or to the growth area limitations. Moreover, since we observed no effect on T24 cell proliferation when cultured with irradiated CFPAC-1, we could exclude the possibility that the effect may be due to radiation-induced bystander effects. Also the results obtained with co-culture of T24 with Saos-2 cells (especially when culture for 24 h or at low ratio), confirm that the decrease in proliferation of T24 cells in presence of hAM-derived cells is not due to a simple decreased surface area available for cell growth or to the irradiation process. When T24 cells were cultured in contact with Saos-2 cells for 48 h and at a ratio of 1:4, or when T24 cells were cultured with skin-derived human fibroblasts, we observed a reduction of their proliferation, indicating that the anti-proliferative effects of hAM-derived cells are in common with other cell types of mesodermal origin. The inhibitory effects of human fibroblasts are not surprising considering that they share immunosuppressive properties similar to mesenchymal stromal cells (Haniffa et al., 2007). Moreover, our findings are in accordance with those of other research showing that that normal fibroblasts inhibit cancer cell proliferation and metastasis of human prostate cancer cells (Iacopino et al., 2012; Koh et al., 2019). Namely, Alkasalias et al. (2014) demonstrated that inhibition is due to two different sets of molecules: (a) the ECM and other surface proteins of fibroblasts which caused contact-dependent inhibition of tumor cell proliferation and (b) the soluble factors secreted by fibroblasts. However, they showed that conditioned media obtained from fibroblasts alone did not inhibit tumor cell proliferation and motility (Alkasalias et al., 2014). Whether or not hAMSC, skin-derived human fibroblasts and, partially, Saos-2 cells adopt the same mechanism of inhibition of T24 cell proliferation needs to be addressed. Concerning the possible mechanism used by hAM-derived cells, Magatti et al. (2012) showed that hAMSC induce cell cycle arrest in hematopoietic and non-hematopoietic cancer cells (Magatti et al., 2012) and Bu et al. (2017) demonstrated that hAEC induce cell cycle arrest in epithelial ovarian cancer cells (Bu et al., 2017). In detail, Magatti et al. demonstrated that the cell cycle arrest is induced by down-regulation in the expression of positive regulators of the cell cycle (cyclins D2, E1, H; cyclin-dependent kinases-2, -4, -6; mini-chromosome maintenance complex, proliferating cell nuclear antigen) and upregulation of the cell cycle inhibitors (G2 cyclin, CDK inhibitor 1A, CDK inhibitor N2B). Furthermore, hAMSC also lead to upregulation of the retinoblastoma protein (pRB) and downregulation of Cullin-1 and RB-1-like protein (p107). Altogether, hAMSC resulted in cycle arrest of cancer cells in the G_0_/G_1_ phase and prevention of cell cycle progression to S phase (Magatti et al., 2012). Similarly, Bu et al. showed that there was an increase in expression of negative regulators of cell cycle progression, namely p16INK4A and p21 and also demonstrated that TGF-β1 secreted from hAEC plays an important role in cell cycle arrest (Bu et al., 2017). Additionally, Niknejad et al. have also shown that cell cycle inhibition can be also caused by inhibition of the heat shock protein 90 (HSP90) (Niknejad et al., 2013b). To conclude, these studies indicate that a decrease in proliferation of T24 cells, grown in co-culture with hAM-derived cells, could be connected with the cell cycle arrest, but this requires further investigation.

Further, to address if the anti-proliferative effect on T24 was mediated by secreted factors, we prevented the cell contact between T24 and hAM-derived cells through the use of transwell system. In this experimental setting, we did not observe a significant decrease of T24 proliferation, suggesting the need of cell-contact in the hAM-derived cells reduction of T24 cell proliferation. These results are in accordance with previously reported data, showing only a mild attenuation of cell growth of breast cancer cells induced by human umbilical cord MSC when cultured in transwell system, compared to the predominantly effect observed in direct cell-contact (Chao et al., 2012), or with studies using the conditioned medium from human WJ-MSC and reported to be unaffected on the proliferation of A549 lung cancer cells (Hendijani et al., 2015) and SPC-A-1 lung adenocarcinoma cells (Meng et al., 2019).

### hAM Scaffolds Alter the Growth Dynamics and Reduce Proliferation of Muscle-Invasive Bladder Cancer Cells

To test whether hAM as a whole, i.e., hAM extracellular matrix with hAM cells, also has anticancer effects, we seeded cancer urothelial T24 cells on the hAM scaffolds. Our results show that T24 cells possess an altered growth dynamic when seeded on hAM scaffolds. Furthermore, in concordance with results of growth dynamics, we have shown that hAM scaffolds diminish the proliferative potential of T24 cells.

The growth-inhibitory effect of hAM scaffolds was most prominent in the first 3 days after seeding and it subsequently decreased. However, it is important to point out that the beneficial effects of hAM scaffolds persisted for at least 3 weeks after seeding (e.g., effects of hAM on morphology and ultrastructure of T24 cells). We assume that most of the various molecules responsible for the anticancer effect were rinsed and thus removed from the hAM scaffolds in the first days of culture, first due to rinsing with PBS during the preparation of scaffolds, and then during replenishing of culture medium during culture on the scaffolds. Moreover, this could also explain why d-hAM scaffolds had the lowest effect on the T24 cells since the preparation of d-hAM scaffolds includes additional rinsing steps due to the treatment with thermolysin and additionally, also depletion of hAEC in d-hAM scaffolds could contribute to lessened growth-inhibitory effect of d-hAM scaffolds.

### hAM Scaffolds Hinder the Invasive Potential of Bladder Cancer Cells

The T24 cell line (ATCC, HTB-4) is derived from a patient with a transitional cell carcinoma of the urinary bladder. This muscle-invasive cell line has been extensively used in bladder cancer research and several studies evaluated its invasiveness (Gildea et al., 2000; Fujiyama et al., 2001; Karam et al., 2011; Hong et al., 2013; Ramakrishnan et al., 2018; Pang et al., 2019). For example, Hong et al. (2013) and Pang et al. (2019) demonstrated that the T24 cells are able to penetrate the extracellular matrix in a Transwell cell invasion assay using Matrigel. Furthermore, Pang et al. (2019) showed that mice, injected with T24 cells form lung metastases and Karam et al. (2011) used the T24 cells to develop a bladder cancer model in mice, which developed primary tumors and subsequent metastases. Moreover, our preliminary data show that the T24 cells are capable of extracellular matrix degradation in scaffolds with or without the appropriate tissue architecture. Namely, the T24 cells invade Matrigel and also the porcine urinary bladder muscle explant culture (data not shown). However, here we presented data, which unequivocally show that when the T24 cells are grown on the hAM scaffolds, even after 3 weeks in the culture, they are not able to cross basal lamina and invade into hAM stroma, despite their potent muscle-invasive capability.

In solid cancers local invasion and metastasis account for more than 90% of mortality (Fernandes et al., 2015). Furthermore, the treatment of solid cancers should be complemented with drugs that inhibit the ability of cancer cells to invade through extracellular matrix (Gandalovičová et al., 2017). Recently, a group of scientists proposed the term “migrastatics” to describe the drugs that interfere with all modes of cancer cell invasiveness and their ability to metastasize (Gandalovičová et al., 2017; Rosel et al., 2019). Moreover, they emphasized the importance of targeting cells migration and/or invasion, since drug resistance, which is the main cause of treatment failure, most often develops due to mutations of the target (Gandalovičová et al., 2017). Therefore, the ability of hAM to prevent disintegration of basal lamina and invasion into hAM stroma is of the utmost importance.

Furthermore, as the expression of N-cadherin is a hallmark of muscle-invasive cancer cells, we evaluated its expression in T24 cells grown on hAM scaffolds and the synthetic scaffolds after 3 weeks in culture. Our results show that hAM scaffolds diminish the expression of N-cadherin in T24 cells, as only a few of the T24 cells grown on hAM scaffolds expressed N-cadherin, while all T24 cells grown on the synthetic scaffolds expressed N-cadherin. Furthermore, even though N-cadherin is junctional protein and is typically expressed in lateral plasma membrane of adjacent cells, in most T24 cells grown on hAM scaffolds, its expression was mostly present in the cytoplasm, and not at the cell periphery.

It is known that N-cadherin is required for pro-migratory signaling and cell-cell adhesion between invasive cells (Sanders et al., 1999; Shih and Yamada, 2012). Shih and Yamada (2012) have shown that the N-cadherin depleted cells migrated more slowly and traveled a shorter distance than wild-type single and clustered cells in a 3D matrix. Moreover, cells with a partial knockdown of endogenous N-cadherin (90% reduction) were still able to migrate at a similar speed to wild-type cells but did not migrate as far. These results indicate that N-cadherin expression is required for the development of migratory cell phenotype, in addition to the formation of cell-cell contacts, and that the level of N-cadherin is critical for promoting the formation of mechanically resilient cell-cell contacts and the migration potential (Shih and Yamada, 2012). Therefore, targeting N-cadherin is a promising therapeutic approach as its downregulation leads to significant inhibition of proliferation, migration and invasion (Bremmer et al., 2015; Gao et al., 2019), which correlates with our own results as well even after 3 weeks in culture.

Snail and Slug are zinc-finger transcription factors that serve as suppressors of E-cadherin transcription and promote EMT (Yu et al., 2010; Wu et al., 2013; Ganesan et al., 2016). Furthermore, their overexpression is linked with tumor progression, invasiveness and poor survival (Bolós et al., 2003; Yu et al., 2010; Nomura et al., 2013; Salehi et al., 2017). The inhibition of Snail, Slug or Twist through siRNA or antisense transfer leads to inhibition of tumor metastasis or growth inhibition and increased susceptibility to chemotherapeutic agents (Vannini et al., 2007; Zhang et al., 2008, 2010), which demonstrates the potential of manipulating EMT markers to achieve a better outcome in bladder cancer treatment. Our results show diminished expression of Snail and Slug in the T24 cells grown on the hAM scaffolds in comparison to the T24 cells grown on synthetic scaffolds and taken together with the results of diminished expression of N-cadherin, collagen IV immunostaining and histological staining, we suggest that hAM harbors anti-cancer molecules that hinder the invasive potential of cancer cells.

Importantly, we have also shown that individual T24 cells grown on hAM scaffolds expressed adherence junctional protein E-cadherin and occludin, one of the key components of tight junctions, which are otherwise not expressed by the T24 cells. Furthermore, T24 cells grown on hAM scaffolds were more tightly attached to each other when grown on the hAM scaffolds and also individual T24 cells on the hAM scaffolds developed some signs of the apico-basal polarity. Therefore, these changes in ultrastructure could be associated with cell cycle arrest and also decreased migration and invasion potential of muscle-invasive bladder cancer cells.

### The Potential of hAM Scaffolds for Use in Regenerative and Reconstructive Urology

Radical cystectomy is the standard of care for muscle-invasive bladder cancer. Besides being associated with considerable morbidity and mortality, this procedure also results in a lower quality of life. New approaches allowing organ preservation are being developed, most of them including chemotherapy and radiation after aggressive transurethral tumor resection (Chen et al., 2013; Mak et al., 2014; Zhong et al., 2019).

We believe it is crucial that new approaches for treatment of bladder cancer are directed also toward mechanisms related to motility, migration and/or invasion and metastasis. Not only hAM limits proliferation and invasion, our group has also shown that hAM is a suitable scaffold for urothelium regeneration since it promotes the proliferation and differentiation of normal urothelial cells (Jerman et al., 2014). Moreover, the urothelium established on s-hAM scaffolds has molecular and ultrastructural properties comparable with the native urothelium (Jerman et al., 2014). Furthermore, the suitability of hAM for bladder reconstruction has been shown *in vivo* by several researchers, used hAM for augmentation after partial cystectomy in rat and dog models. Importantly, researchers report the successful regeneration of bladder mucosa and neovascularization of bladder tissue (Iijima et al., 2007; Shakeri et al., 2008; Adamowicz et al., 2016).

To conclude, our results show for the first time that hAM scaffolds have adverse effects on cancer urothelial cells and their invasiveness. Therefore, further studies are needed to evaluate the possibility of using hAM in the treatment of bladder cancer as a post-operative wound dressing, which would diminish adhesion of cancer urothelial cells to the exposed lamina propria, their proliferation and invasion, as well as promote the rapid regeneration of still normal and healthy urothelium.

## Data Availability Statement

The authors declare that all data supporting the findings of this study are available within the article. All information about the Materials and Methods used are available also in the Protocols.io database (Ramuta et al., 2020a).

## Ethics Statement

The studies involving human participants were reviewed and approved by National Medical Ethics Committee of the Republic of Slovenia (decree numbers 43/12/09 and 0120-179/2018/5), and Comitato Etico Provinciale of Brescia number NP 2243 (19/01/2016), Italy. The patients/participants provided their written informed consent to participate in this study. The animal study was reviewed and approved by The experiments were approved by the Veterinary Administration of the Slovenian Ministry of Agriculture and Forestry in compliance with the Animal Health Protection Act and the Instructions for Granting Permits for Animal Experimentation for Scientific Purposes.

## Author Contributions

TZR, UDJ, LT, AJ, MM, EV, PBS, and MK performed the experiments. TZR, UDJ, LT, AJ, MM, EV, PBS, ARS, OP, and MK interpreted the results, reviewed and edited the manuscript, and approved the final manuscript. TZR wrote the first draft of the manuscript. All authors contributed to the article and approved the submitted version.

## Conflict of Interest

The authors declare that the research was conducted in the absence of any commercial or financial relationships that could be construed as a potential conflict of interest.

## Footnotes

1 ClinicalTrials.gov

## Abbreviations

d-hAM: hAM scaffold, in which the epithelium was removed (deepithelized hAM); the T24 cells were seeded on the basal lamina
e-hAM: hAM scaffold; the T24 cells were seeded on the hAM epithelium
hAEC: human amniotic epithelial cells
hAM: human amniotic membrane
hAMSC: human amniotic mesenchymal stromal cells
s-hAM: hAM scaffold; the T24 cells were seeded on the hAM stroma.
